# Assessment of Physical/Mechanical Performance of Dental Resin Sealants Containing Sr-Bioactive Glass Nanoparticles and Calcium Phosphate

**DOI:** 10.3390/polym14245436

**Published:** 2022-12-12

**Authors:** Piyaphong Panpisut, Nannapat Praesuwatsilp, Phubet Bawornworatham, Parichart Naruphontjirakul, Somying Patntirapong, Anne M. Young

**Affiliations:** 1 Faculty of Dentistry, Thammasat University, Pathum Thani 12120, Thailand; 2 Thammasat University Research Unit in Dental and Bone Substitute Biomaterials, Thammasat University, Pathum Thani 12120, Thailand; 3 Biological Engineering Program, Faculty of Engineering, King Mongkut’s University of Technology Thonburi, Bangkok 10140, Thailand; 4 Division of Biomaterials and Tissue Engineering, UCL Eastman Dental Institute, Royal Free Hospital, Rowland Hill Street, London, NW3 2PF, UK

**Keywords:** pit and fissure sealants, resin sealant, strontium, bioactive glass, monocalcium phosphate monohydrate, polymerization, biaxial flexural strength, calcium phosphate, mechanical properties

## Abstract

The aim of this study was to assess the chemical/mechanical properties of ion-releasing dental sealants containing strontium-bioactive glass nanoparticles (Sr-BGNPs) and monocalcium phosphate monohydrate (MCPM). Two experimental sealants, TS1 (10 wt% Sr-BGNPs and 2 wt% MCPM) and TS2 (5 wt% Sr-BGNPs and 4 wt% MCPM), were prepared. Commercial controls were ClinproXT (CP) and BeautiSealant (BT). The monomer conversion (DC) was tested using ATR–FTIR (n = 5). The biaxial flexural strength (BFS) and modulus (BFM) were determined (n = 5) following 24 h and 7 days of immersion in water. The Vickers surface microhardness (SH) after 1 day in acetic acid (conc) versus water was tested (n = 5). The bulk and surface calcium phosphate precipitation in simulated body fluid was examined under SEM-EDX. The ion release at 4 weeks was analyzed using ICP-MS (n = 5). The DC after 40 s of light exposure of TS1 (43%) and TS2 (46%) was significantly lower than that of CP (58%) and BT (61%) (*p* < 0.05). The average BFS of TS1 (103 MPa), TS2 (123 MPa), and BT (94 MPa) were lower than that of CP (173 MPa). The average BFM and SH of TS1 (2.2 GPa, 19 VHN) and TS2 (2.0 GPa, 16 VHN) were higher than that of CP (1.6 GPa, 11 VHN) and BT (1.3 GPa, 12 VHN). TS1 showed higher Ca, P, and Sr release than TS2. Bulk calcium phosphate precipitation was detected on TS1 and TS2 suggesting some ion exchange. In conclusion, the DC of experimental sealants was lower than that of commercial materials, but their mechanical properties were within the acceptable ranges. The released ions may support remineralizing actions.

## 1. Introduction

Untreated dental caries is the most common preventable chronic disease. The current trend for caries management emphasizes prevention and control of active lesions instead of focusing on irreversible surgical management [1]. Dental sealants have been reported to be an effective and cost-effective method for arresting non-cavitated occlusal caries [2,3]. Resin sealant is the most used pit and fissure sealing material. This is due mainly to command setting upon light-exposure. The high failure rate of resin sealants, however, has been a major concern. In one study, 27.7% of resin sealants were observed to fail within 1 year [4]. Common reasons for failure are loss of retention, marginal leakage, and secondary caries, especially in high-caries-risk groups [5] or patients with limited compliance [6].

The addition of fillers that can release ions, such as calcium and phosphate ions, was expected to help promote ion saturation suitable for mineral precipitation to prevent secondary caries around dental sealants [7]. Previous studies have demonstrated that the addition of monocalcium phosphate monohydrate (MCPM) promoted the release of calcium and phosphate ions. These could enhance mineralizing actions of resin composite restoratives [8,9,10,11]. Additionally, it was reported that MCPM can absorb and react with residual surface water [12], which may aid sealant bonding. Bonding may be further improved through the addition of monomers with acidic groups such as 10-methacryloyloxydecyl dihydrogen phosphate (10-MDP) [13]. Previous studies suggest that this may additionally act as a coupling agent between the MCPM and the resin phase, thereby also improving the mechanical properties [14]. 

Other studies have reported that the addition of bioactive glass nanoparticles (BGNPs) could also potentially promote remineralizing, in addition to their antibacterial actions for resin-based materials [15,16,17]. The use of Sr-BGNPs promoted Ca, P, and Sr ions, which are beneficial for acid neutralization and remineralizing effects. The previously developed resin composites for orthodontic adhesives also showed that the addition of Sr-BGNPs could inhibit planktonic *S. mutans* growth [18]. It was proposed that the antibacterial actions of bioactive glass may be due to the release of ions that increase osmotic pressure, thus producing unsuitable conditions for bacterial growth. It was expected that the replacement of SrO by CaO in the glass network might increase the surface reactivity of the glass [19], which could potentially promote ion release. Furthermore, it was hypothesized that the addition of calcium phosphates, such as MCPM, could additionally promote water sorption that could enhance the reactivity of bioactive glass. The preparation of dental sealant containing both Sr-BGNPs and MCPM has not yet been investigated. The increase in ion release may subsequently promote remineralizing actions for resin-based dental sealant. 

The addition of calcium phosphates may, however, increase light scattering. This could reduce the degree of monomer conversion achieved through light activation and thereby mechanical properties [10]. The aim of this study was, therefore, to prepare experimental resin sealants with added Sr-bioactive glass nanoparticles (Sr-BGNPs) and monocalcium phosphate monohydrate (MCPM). The degree of monomer conversion, biaxial flexural strength/modulus, surface microhardness, calcium phosphate precipitation, and ion release was examined and compared with commercial materials. The null hypothesis is that the experimental materials exhibit no significant difference in chemical and mechanical properties compared with commercial materials. 

## 2. Materials and Methods

### 2.1. Preparation of Experimental Dental Sealants 

The experimental resin sealants were prepared using a powder to liquid ratio of 1:1 (mass ratio). The liquid phase contained 70 wt% urethane dimethacrylate (UDMA, Sigma-Aldrich, St. Louis, MO, USA), 23 wt% triethyleneglycol dimethacrylate, 1 wt% camphorquinone (CQ, Sigma-Aldrich, St. Louis, MO, USA), 3 wt% 10-MDP (Watson international Ltd., London, UK), and 3 wt% 2-hydroxyethyl methacrylate (HEMA, Sigma-Aldrich, St. Louis, MO, USA). The liquid phase was mixed in an amber bottle with a magnetic stirrer over a hotplate. 

**Figure 1:**
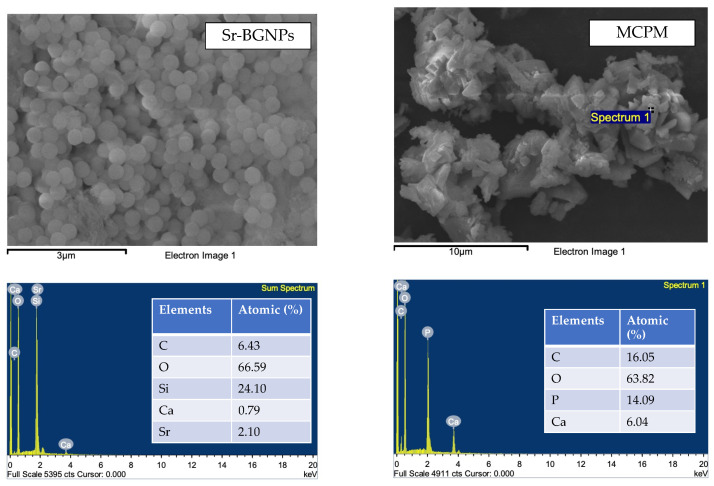
SEM images with EDX data of Sr-BGNPs and MCPM.

The powder phase contained boroaluminosilicate glass (non-silanated 180 nm and silanated 0.7 μm with 1:1 mass ratio, Esstech Inc., Essington, PA, USA), Sr-containing bioactive glass nanoparticles (Sr-BGNPs, King Mongkut ’s University of Technology Thonburi, Bangkok, Thailand), and monocalcium phosphate monohydrate (MCPM, Himed, Old Bethpage, NY, USA). Two formulations of experimental dental sealants were prepared (Table 1). The scanning electron microscope (SEM) images with energy-dispersive X-ray (EDX) data of filler additives are provided in Figure 1. Sr-BGNPs were prepared using the sol–gel technique according to the protocol used in a previous study [18]. 

The liquid and powder phases were weighed using a four-figure balance (MS-DNY- 43, METTLER TOLEDO, Columbus, OH, USA). They were hand-mixed using a plastic spatula. The mixed paste was loaded into a composite syringe (medmix Switzerland AG, Baar, Switzerland). The commercially available pit and fissure resin sealants (Clinpro^TM^ Sealant and BeautiSealant) were used as the commercial controls (Table 2). 

### 2.2. Degree of Monomer Conversion (DC)

The degree of monomer conversion was assessed using ATR–FTIR (Nicolet IS5, Thermo Fisher Scientific, Waltham, MA, USA). The test was performed at room temperature (25 ± 1 °C). The materials were injected into a metal ring (1 mm in thickness and 10 mm in diameter) (n = 5) placed around the ATR diamond. They were covered with an acetate sheet and light cured for 20 s or 40 s using an LED light-curing unit (1250 mW/cm^2^, Smartlite Focus, DENTSPLY Sirona, York, PA, USA) [20]. The curing-light position was fixed at ~2 mm from the surface of the specimen. The FTIR spectra of the specimens between 700 and 4000 cm^−1^, before and after curing, were recorded. The degree of monomer conversion (DC) of the sealants was calculated using the following equation:

(1) $$\mathrm{DC}\left(\%\right)=\frac{100\left(B_{0}-B_{t}\right)}{B_{0}}$$

where B_0_ and B_t_ are the absorbance of the C-O peak (1320 cm^−1^) [21] above the background level at 1335 cm^−1^ initially and after time *t*. This C-O bond lengthens and shifts to lower wavenumbers (1250 cm^−1^) when the adjacent C=C group reacts. The C-O peak was used instead of the C=C peak at 1640 cm^−1^ as it has been shown to provide more reproducible results [18].

### 2.3. Biaxial Flexural Strength (BFS) and Modulus (BFM)

BFS and BFM were examined using a ball-on-ring testing jig under the mechanical testing frame (AGSX, Shimadzu, Kyoto, Japan). Disc specimens were prepared by injecting the materials into a metal ring (1 mm in thickness and 10 mm in diameter) (n = 5). The specimens were covered with an acetate sheet and light-cured using a circular motion for 40 s on the top and bottom surfaces. They were left at room temperature for 24 h. Then, they were removed from the rings and immersed in a tube containing 5 mL of artificial saliva and incubated at 37 °C for 24 h or 7 days. Prior to the test, the specimen’s thickness was measured using a digital vernier caliper (YOUFOUND, FISCO, Yokohama, Japan). The discs were placed on the ball-on-ring testing jig under the mechanical testing frame. The specimens were loaded with a 500 N load cell with a crosshead speed of 1 mm/min. The biaxial flexural strength (BFS, Pa) was obtained using the following equation [10]:

(2) $$\mathrm{BFS}\;=\frac{F}{d^{2}}\{\left(1+v\right)\left[0.485\ln\left(\frac{r}{d}\right)+0.52\right]+0.48\}$$

where F is the load at failure (N), d is the specimen’s thickness (m), r is the radius of circular support (m), and v is Poisson’s ratio (0.3). Additionally, the biaxial flexural modulus (BFM) was calculated using the following equation.

(3) $$\mathrm{BFM}\;=\left(\frac{\Delta H}{\Delta W_{c}}\right)\times \left(\frac{\beta_{c}d^{2}}{q^{3}}\right)$$

where $\frac{\Delta H}{\Delta W_{c}}$ is the rate of change of load with regard to central deflection (N/m), $\beta_{c}$ is the center deflection junction (0.5024) [22], and q is the ratio of support radius to the radius of the disc. 

### 2.4. Vickers Surface Microhardness

Surface microhardness upon immersion in water or acid were examined using a microhardness tester (FM-800, Future-Tech Corp, Kanagawa, Japan). Disc specimens were prepared (0.5 mm in thickness and 5 mm in diameter) (n = 5). The Vickers surface microhardness was measured at the surface of the specimens before and after immersion in 2 mL of deionized water and acetic acid (0.2 mM) at 24 h. The test was conducted using a microhardness tester at a load of 300 g with a loading time of 10 s [23]. Four readings for each specimen were recorded and reported as Vickers hardness number (VHN). 

### 2.5. Surface Calcium Phosphate Precipitation 

The precipitation of calcium phosphate was determined using a scanning electron microscope (SEM, JSM, 7800F, JEOL Ltd., Tokyo, Japan) equipped with energy-dispersive X-ray (EDX, Inca X-sight 6650 detector, Oxford Instruments, Abingdon, UK). Disc specimens were prepared (1 mm in thickness and 10 mm in diameter) (n = 1) and immersed in 5 mL simulated body fluid (SBF) prepared according to ISO BS ISO 23317:2014 [24]. The specimens were incubated at 37 °C for 7 days. Then, they were removed, blotted dry and sputter coated with Au. The surface of specimens was examined using a SEM and EDX with a beam voltage set at 10 kV.

### 2.6. Ion Release 

The ion release was analyzed using an inductively coupled plasma mass spectrometer (ICP-MS, NexION 350X, PerkinElmer, Waltham, MA, USA). The specimens were prepared and immersed in 5 mL of deionized water for 4 weeks (n = 5). The solution was collected to analyze the concentration of ions (Ca, P, and Sr ions) using ICP-MS with the KED (kinetic energy discrimination) mode. The calibration was performed using instrument calibration standard 2 (PerkinElmer, Waltham, MA, USA). Data were analyzed using Syngistix for ICP-MS version 1.0. 

### 2.7. Statistical Analysis 

Data were analyzed using IBM SPSS Statistics version 27 for macOS (SPSS Inc., Chicago, IL, USA). The normality of the data was examined using a Shapiro–Wilk test. For normally distributed data, one-way ANOVA followed by Tukey posthoc multiple comparisons was used. For non-normally distributed data, Kruskal–Wallis, followed by the Dunn test was used. A paired t-test was employed to compare the DC after light-curing for 20 and 40 s. The significance value was set at *p* = 0.05. Additionally, a posthoc power analysis was performed, using G*Power version 3.1.9.6 (Heinrich-Heine-Universität Düsseldorf, Düsseldorf, Germany) [25], using the data from previously published studies that developed and tested similar materials [11,20]. The analysis indicated that the sample size used in each test exhibited a power > 0.95 at alpha = 0.05 for a one-way ANOVA.

## 3. Results

### 3.1. Degree of Monomer Conversion 

**Figure 2:**
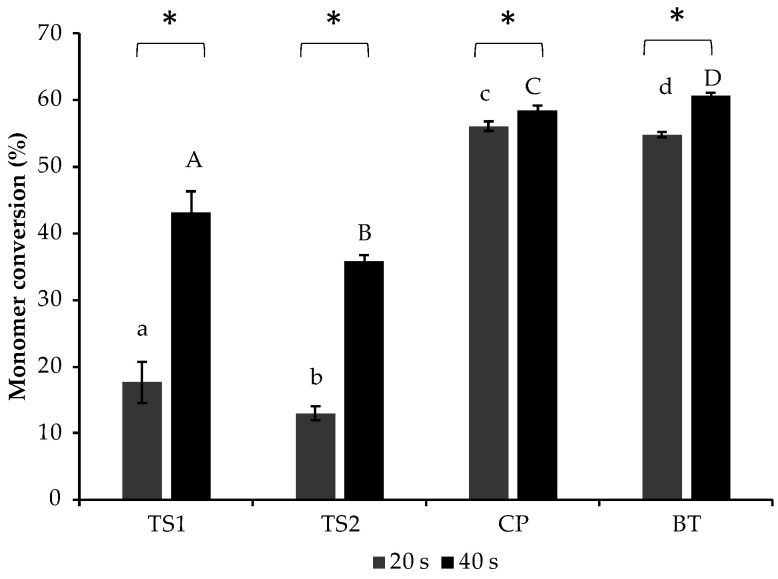
Degree of monomer conversion after light-curing for 20 s and 40 s. Different lower-case and upper-case letters indicate significant differences (*p* < 0.05) among materials after light-curing for 20 s and 40 s, respectively. Stars (*) indicate significant differences (*p* < 0.05) within the same materials. Error bars are SD (n = 5).

The highest and lowest DCs after curing for 20 s were obtained from CP (56.1 ± 0.8%) and TS2 (13.0 ± 1.0%) (Figure 2). The DC of TS2 was comparable to that of TS1 (17.6 ± 3.2%) (*p* = 0.117). The DCs of both TS1 and TS2 were significantly lower than that of CP or BT (54.8 ± 0.4%) (*p* < 0.05). After curing for 40 s, the DCs of all materials were significantly increased. This was clearly observed with TS1 and TS2. The DCs, at 40 s, of TS1 (43.1 ± 2.5%) and TS2 (35.8 ± 2.6%) were also significantly lower than that of CP (58.4 ± 0.9%) or BT (60.8 ± 0.5%) (*p* < 0.05).

### 3.2. Biaxial Flexural Strength (BFS) and Biaxial Flexural Modulus (BFM) 

**Figure 3:**
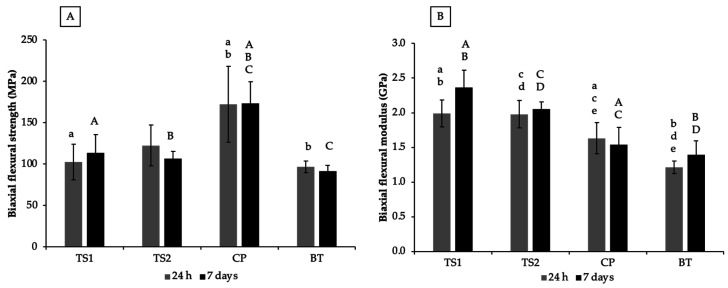
(**A**) BFS and (**B**) BFM of materials after immersion for 24 h and 7 days. The same lower-case and upper-case letters indicate significant differences (*p* < 0.05) among materials at 24 h and 7 days, respectively. Error bars are SD (n = 5).

The highest BFS at 24 h was detected with CP (172 ± 46 MPa), which was significantly higher than that of TS1 (103 ± 22 MPa) (*p* = 0.0068) and BT (97 ± 7 MPa) (*p* = 0.0035) (Figure 3). The BFS of TS1 was comparable to that of TS2 (123 ± 25 MPa) (*p* = 0.796) and BT (*p* = 0.993). At 7 days, a significant reduction in BFS was not detected. The BFS of CP (173 ± 26 MPa) was significantly higher than that of TS1 (113 ± 23 MPa) (*p* = 0.0004), TS2 (106 ± 9 MPa) (*p* = 0.0001), and BT (92 ± 7 MPa) (*p* < 0.01). 

For BFM, the highest and lowest values at 24 h were detected with TS1 (1.99 ± 0.19 GPa) and BT (1.21 ± 0.09 GPa). The BFM of TS2 was similar to that of TS1 (1.98 ± 0.20) (*p* = 0.999) but the values of both formulations were significantly higher than that of CP (1.63 ± 0.22) and BT (1.21 ± 0.09) (*p* < 0.05). The BFM at 7 days of TS1 (2.36 ± 0.25) was similar to that of TS2 (2.05 ± 0.11 GPa) (*p* > 0.99). The BFM values of TS1 and TS2 at 7 days were significantly higher than that of CP (1.54 ± 0.25 GPa) and BT (1.39 ± 0.20 GPa) (*p* < 0.05). 

**Figure 4:**
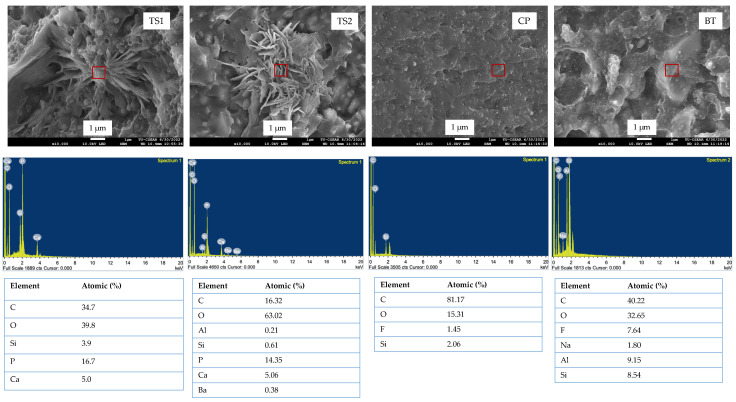
SEM images with EDX results of the fracture surface of the representative specimens after BFS testing at 7 days.

The fracture surface after BFS testing at 7 days revealed the precipitation of calcium phosphate inside TS1 and TS2 (Figure 4). CP showed a smoother fracture surface compared with other materials. Fluoride was detected on the fracture surface of CP and BT. 

### 3.3. Vickers Surface Microhardness (SH) 

**Figure 5:**
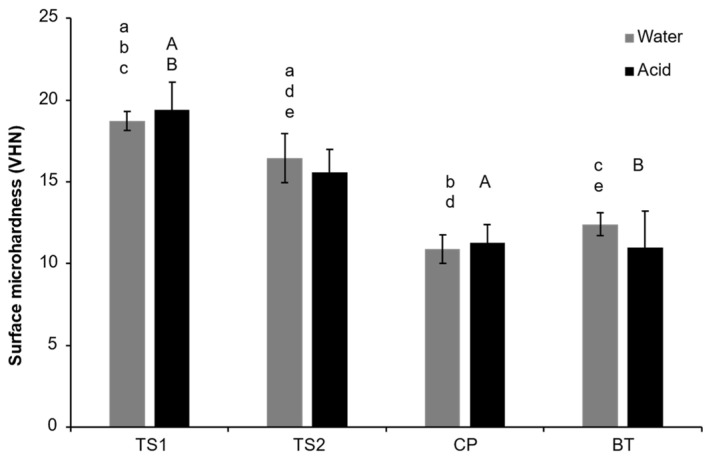
Surface microhardness after immersion in water and acetic acid (0.2 mM) for 24 h. The same lower-case and upper-case letters indicate significant differences (*p* < 0.05) between materials in acid and water, respectively. Error bars are SD (n = 5).

The highest and lowest SHs in water were detected with TS1 (18.7 ± 0.6 VHN) and CP (10.9 ± 0.9 VHN) (Figure 5). The SH of TS1 was significantly higher than that of TS2 (16.6 ± 1.5 VHN). Additionally, TS1 showed significantly higher SH than that of CP or BT (12.4 ± 0.7 VHN) (*p* < 0.05).

Following acid immersion (0.2 mM acetic acid), the SH of TS1 (19.4 ± 1.7 VHN) was significantly higher than that of CP (11.3 ± 1.1 VHN) or BT (11.0 ± 2.2 VHN) (*p* < 0.05). The SH of TS1 after immersion in the acid was similar to that of TS2 (15.6 ± 1.4 VHN) (*p* = 1.000). Additionally, no significant difference in SH for immersion in water or acid was detected in any material.

### 3.4. Surface Calcium Phosphate Precipitation 

**Figure 6:**
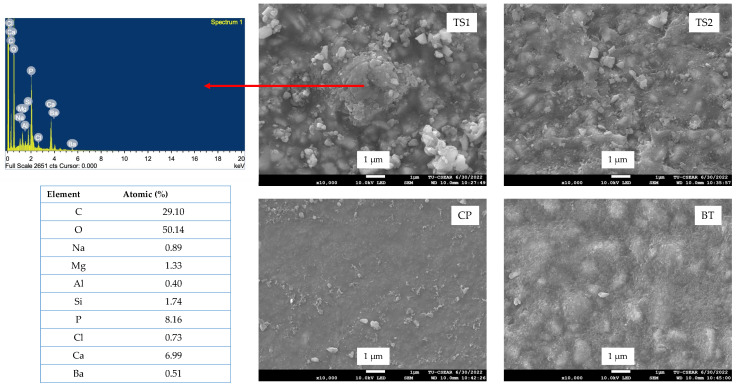
SEM images with EDX results of the specimen surface after immersing in SBF for 7 days.

No precipitation was detected on the surface of the BT and CP specimens, as was expected. The precipitation of calcium phosphate was slightly detected on the surface of TS1 (Figure 6). However, no precipitation was observed on TS2. The EDX results indicated that the precipitate on TS1 contained Ca and P. 

### 3.5. Ion Release

BT exhibited a significantly higher Ca release than TS2 (*p* = 0.002) and CP (*p* < 0.01) (Table 3). TS1 showed comparable Ca ion release to BT (*p* = 0.995), TS2 (*p* = 0.536), and CP (*p* = 0.155). For P release, the value obtained from BT was lower than the detection limit of the instrument. The P release of TS1 was comparable to TS2 (*p* = 0.927). The P release of both TS1 and TS2 was significantly higher than that of CP (*p* < 0.05). The Sr release from TS1 was similar to that of TS2 (*p* = 0.187) and BT (*p* = 1.00). That from CP was lower than the instrument detection limit. 

## 4. Discussion

The objective of this study was to prepare ion-releasing resin sealants through the addition of Sr-BGNPs and MCPM. Their degree of monomer conversion (DC), biaxial flexural strength/modulus (BFS/BFM), Vickers surface microhardness (SH) and surface calcium phosphate precipitation were compared with two commercial materials. Despite release of ions and slow monomer conversion, the mechanical strengths of TS1 and TS2 were not significantly different from that of BT. With future modifications, strengths could potentially be further improved. Changes could include silanation of all particles and/or MDP optimization. Additionally, reduced air-bubble incorporation with vacuum and/or centrifugal mixing of the paste might be beneficial [12]. 

The degree of monomer conversion (DC) affects the physical/mechanical performance of light-activated resin-based materials [26]. The DC obtained from resin-based materials can vary from 35 to 77%, which is within the range of DC reported in a published study [27]. The results from the current study suggested that TS1 and TS2 required a curing time greater than 20 s to enable a DC comparable with commercial formulations. DC of the experimental dental sealants after at both 20 and 40s was much lower than the commercial materials. Low curing could be due to the additives (Sr-BGNPs and MCPM) which may increase light-scattering and limit light transmission to the bottom surface of the materials. Additionally, the CQ could limit light transmission. In future work, transmission and conversion rate might be improved through replacement of some of the CQ by alternative initiators and/or an amine coactivator [28]. The increase in curing time may increase the formation of polymers in the experimental sealants. This may reduce the monomer/filler refractive index mismatch on the top surface [29,30] and enable light transmission into the specimen.

The DC of experimental resin sealants at 40 s was still lower than the DC of the commercial materials. It was speculated that the high DCs of CP and BT could be due to a higher percentage of low molecular weight monomers such as TEGDMA (~50 wt%) compared with the experimental materials (~25 wt%). The use of TEGDMA (glass transition temperature = −82 °C, molecular weight = 286 g/mol, viscosity = 0.05 Pa·s) may help reduce the viscosity of resin sealant [31]. This may promote the adaptation of materials on irregular deep pits and fissures of the occlusal surface. It is known that low molecular weight monomers with low glass transition temperature increased the mobility of the polymerizing mixture [32,33], thus enhancing polymerization of the materials. The low molecular weight of TEGDMA, however, causes high shrinkage. Alternative higher molecular weight diluents such as poly (propylene glycol) dimethacrylate could help to reduce this issue and further improve conversion [32,34]. Additionally, activators or tertiary amines contained in CP (EDMAB) and BT (DMAEMA) may help to increase the degree of monomer conversion of the materials [35]. 

A major concern with the experimental resin sealants is the risk of releasing toxic monomers due to their low DC. Therefore, the monomer phase of the experimental sealants should be adjusted in future work to increase their DC. This could be achieved by increasing the proportion of low molecular weight monomers. Another possibility could be the use of a highly reactive photo-initiator such as mono-alkyl phosphine oxide (TPO) with activators [28,36]. The BS EN ISO 6874:2015 requires a depth-of-cure of at least 1.5 mm from the light-activated pit and fissure sealants [37]. Hence, future work should employ depth-of-cure studies. 

The biaxial flexural strength (BFS) and surface microhardness tests were employed to ensure that the materials would provide sufficient mechanical strength and be comparable to the commercial materials. The current study employed BFS testing due to the low variability of the obtained results compared with the traditional 3-point bending test [38]. The biaxial flexural strength and modulus of the TS1 and TS2 were within the range of that observed with CP and BT. This could be due to the fact that the specimens were cured on both top and bottom surfaces of the specimens. This may sufficiently allow adequate light to penetrate into the bulk of the materials. The low biaxial flexural strength of TS1 and TS2 compared with CP could be due to the lack of silanization of the reactive fillers. The use of 3-methacryloxypropyltrimethoxysilane (MPTMS) as a silane coupling agent for reactive fillers may promote dispersion of fillers in the resin matrix. However, the hydrophobicity of silane may also reduce water sorption by the fillers and thereby ion release [39]. The formation of calcium phosphate precipitate inside the materials may suggest that the reactive fillers were reacting with absorbed water leading to dissolution/precipitation of calcium phosphate fillers in the materials. The fillers were expected to prevent cracks, which could potentially help impede and prevent crack propagation of the materials [9]. The smooth fracture surface detected with CP indicated excellent dispersion of fillers in the materials. Additionally, EDX also revealed that the materials contained fluorine, which could be the inorganic fluoride filler (tetrabutylammonium tetrafluoroborate) contained in the material. 

The fillers observed in the fracture surface of BT were expected to be surface pre-reacted glass fillers (S-PRG). The fillers may contain a hydrophilic component, such as a silicious hydrogel phase [40], which may encourage water sorption into the resin matrix. This may subsequently promote acid–base reaction and ion release. However, the excessive sorption of water may cause polymer plasticization, thus reducing the rigidity or modulus of elasticity of the material. It was speculated that a higher level of glass fillers in the experimental materials (~50 wt%) may lead to a greater modulus of elasticity of the materials compared with the commercial materials. The safety data sheets provided from the suppliers indicated that the glass filler CP and BT content was approximately 10 wt% and 30 wt%, respectively. 

High surface microhardness may help reduce the wear of materials upon mastication [41]. The greater glass filler content could also ensure surface hardness remains high even with water and acid storage. This may help to ensure reduced wear upon mechanical abrasion or exposure to acid. The lower surface microhardness of TS2 compared to TS1 could be due to its higher hydrophilic MCPM content. It was speculated that MCPM may encourage water sorption, which could plasticize the polymer matrix, reducing the rigidity of the polymer network at the surface [42]. The remaining of unreacted monomers could also affect the rigidity of the surface [20]. 

The assessment of ion-releasing and calcium phosphate precipitation was expected to confirm the initial desirable mineralizing actions of the materials. No calcium phosphate precipitation was detected on the surface of both CP and BT. The possible explanation could be that CP and BT exhibited a low level of Ca and P release, respectively. However, it should be mentioned that CP and BT contain F which is expected to help repair demineralized enamel via the formation of slightly soluble fluorohydroxyapatite. Additionally, BT contained S-PRG filler, which was expected to control the colonization of cariogenic bacteria in active caries lesions underneath the sealant [43]. TS1 promoted a higher concentration of Ca and Sr release than TS2. This may be due to the higher level of bioactive glass nanoparticles in TS1. The ability of TS1 to promote Ca, P, and Sr ions may enable the saturated condition for calcium phosphate precipitation [44]. However, the level of precipitation was much lower than that previously observed with resin composites containing higher levels of MCPM and TCP. Another possible cause may be the use of a low powder-to-liquid ratio in the current study (PLR 1:1) compared with the previous studies (PLR 4:1 [10] or 2.7:1 [9]). A large variation in the results of Ca and Sr release was observed with the experimental resin sealants. This may be due to the variable dissolution/reprecipitation of calcium phosphate that occurred in the materials. Hence, the result should be confirmed in future testing with a larger sample number. The mineralizing action of the materials was expected to help to reduce marginal leakage and promote the remineralizing actions of the resin sealant. This should be investigated in future work. The beneficial clinical effects of both CP and BT in preventing dental caries still require high-quality clinical evidence. It should be mentioned that the current study is an in vitro study, so the clinical relevance must be interpreted carefully.

## 5. Conclusions

The experimental resin sealants containing Sr-bioactive glass nanoparticles (Sr-BGNPs) and monocalcium phosphate monohydrate (MCPM):

- Required a curing time of 40 s to achieve a high DC;
- Had a BFS that was between the BFSs of commercial resin sealants;
- Had a BFM and SH higher than those of the commercial products;
- Exhibited ion release that could potentially encourage calcium phosphate precipitation and help to reduce secondary caries around the resin sealant.

## 6. Patents

A.M.Y. has one patent on the use of MCPM in dental composites licensed to a dental company (Davis Schottlander and Davis Ltd., Letchworth Garden City, UK).

## Figures and Tables

**Table 1:** Composition of the experimental resin sealant fillers.

Formulations	Sr/F-BGNPs (wt%)	MCPM (wt%)	Glass (wt%)
TS1	10	2	88
TS2	5	4	91

**Table 2:** Components in commercial resin sealants. Only approximate component levels are provided by the manufacturers.

Name	Composition	Manufacturer	Lot No.
Clinpro^TM^ Sealant (CP)	Triethylene glycol dimethacrylate (40–50 wt%), bisphenol A-glycidyl methacrylate (40–50 wt%), silane treated silica (5–10 wt%), tetrabutylammonium tetrafluoroborate (<5 wt%), diphenyliodonium hexafluorophosphate (<1 wt%), triphenylantimony (<0.5 wt%), DL-camphoquinone (<2 wt%, ethyl 4-(dimethylamino)benzoate (EDMAB, <2 wt%), titanium dioxide (<0.5 wt%), hydroquinone (<0.05 wt%)	3M, Saint Paul, MN, USA	09997
BeautiSealant (BT)	Surface pre-reacted glass fillers (S-PRG, 30 wt%), micro fumed silica, urethane dimethacrylate (25–30 wt%), triethyleneglycol dimethacrylate, dimethyl aminoethyl methacrylate (0.1–2 wt%)	SHOFU INC., Kyoto, Japan	092101

**Table 3:** The concentration of released ions (mean ± SD, μg/L) after immersion in water for up to 4 weeks. The same letters within the same column indicated significant differences (*p* < 0.05) (n = 3). “ND” indicated that the value was lower than the detection limit of the instrument.

Formulations	Ca	P	Sr
TS1	1421 ± 1043 (a)	214 ± 54 (a)	1620 ± 1329
TS2	611 ± 310	236 ± 74 (b)	666 ± 358
CP	15 ± 10 (a,b)	24 ± 7 (a,b)	ND
BT	1650 ± 214 (b)	ND	1614 ± 210

## Data Availability

The datasets generated and/or analyzed during the current study are available from the corresponding author on reasonable request.
